# Reference Values of 14 Serum Trace Elements for Pregnant Chinese Women: A Cross-Sectional Study in the China Nutrition and Health Survey 2010–2012

**DOI:** 10.3390/nu9030309

**Published:** 2017-03-21

**Authors:** Xiaobing Liu, Yu Zhang, Jianhua Piao, Deqian Mao, Yajie Li, Weidong Li, Lichen Yang, Xiaoguang Yang

**Affiliations:** Key Laboratory of Trace Element Nutrition of National Health and Family Planning Commission, National Institute of Nutrition and Health, Chinese Center for Disease Control and Prevention, Beijing 10000, China; liuxiaobing009@126.com (X.L.); hmuyufang@163.com (Y.Z.); piaojh@163.com (J.P.); dqmao@126.com (D.M.); yjsome@163.com (Y.L.); WeidongLi57@126.com (W.L.); yanglichen_28@126.com (L.Y.)

**Keywords:** reference values, trace elements, high-resolution inductively coupled mass spectrometry, China Nutrition and Health Survey 2010–2012

## Abstract

The development of reference values of trace elements is recognized as a fundamental prerequisite for the assessment of trace element nutritional status and health risks. In this study, a total of 1400 pregnant women aged 27.0 ± 4.5 years were randomly selected from the China Nutrition and Health Survey 2010–2012 (CNHS 2010–2012). The concentrations of 14 serum trace elements were determined by high-resolution inductively coupled plasma mass spectrometry. Reference values were calculated covering the central 95% reference intervals (P2.5–P97.5) after excluding outliers by Dixon’s test. The overall reference values of serum trace elements were 131.5 (55.8-265.0 μg/dL for iron (Fe), 195.5 (107.0–362.4) μg/dL for copper (Cu), 74.0 (51.8–111.3) μg/dL for zinc (Zn), 22.3 (14.0–62.0) μg/dL for rubidium (Rb), 72.2 (39.9–111.6) μg/L for selenium (Se), 45.9 (23.8-104.3) μg/L for strontium (Sr), 1.8 (1.2–3.6) μg/L for molybdenum (Mo), 2.4 (1.2–8.4) μg/L for manganese (Mn), 1.9 (0.6–9.0) ng/L for lead (Pb), 1.1 (0.3-5.6) ng/L for arsenic (As), 835.6 (219.8–4287.7) ng/L for chromium (Cr), 337.9 (57.0–1130.0) ng/L for cobalt (Co), 193.2 (23.6–2323.1) ng/L for vanadium (V), and 133.7 (72.1–595.1) ng/L for cadmium (Cd). Furthermore, some significant differences in serum trace element reference values were observed between different groupings of age intervals, residences, anthropometric status, and duration of pregnancy. We found that serum Fe, Zn, and Se concentrations significantly decreased, whereas serum Cu, Sr, and Co concentrations elevated progressively compared with reference values of 14 serum trace elements in pregnant Chinese women. The reference values of serum trace elements established could play a key role in the following nutritional status and health risk assessment.

## 1. Introduction

Trace elements are inorganic constituents needed in minute quantities but considered nutrients essential for human health. Both a deficiency and an excessive accumulation can result in multiple physiological dysfunctions [1]. It has been shown that the status of trace elements can reflect total exposure of all possible sources. The determination of specimens in humans is valuable for characterizing the body burden of metals, toxics, and nutrients [2]. Moreover, compared to environmental analyses, it is a more direct way of estimating exposure, as it can distinctly depict where stronger environmental actions or medical decisions are required. Hence, the development of trace element reference values for specific populations has long been considered a prerequisite for assessing the exposure and absorption of trace elements, despite its complexity [3]. 

Pregnancy is a period of increased metabolic demands, mainly due to changes in the woman’s physiology and the requirements of the growing fetus [4]. Oxygen consumption, central hemodynamic alterations, and oxidative stress are altered, contributing to a determination of the long-term health in pregnant women. Some trace element concentrations are also altered during this vulnerable period. Therein, it is worth noting that the deficiency or overexposure of certain trace elements could be detrimental to the health of both pregnant women and their fetuses [5]. Pregnant women are often exposed to inadequate macro- and micronutrition in developing countries or regions. Thus, the establishment of trace element reference values for pregnant women is essential for the assessment of trace element nutrition and potential health risks. 

In general, the levels of trace element uptake are more easily affected by dietary habits, lifestyles, and environmental conditions. There is clearly a difference in trace elements in different countries and regions around the world. Germany [6], the United States [7], Canada [8], and other countries [9,10] have derived reference values of trace elements using their own data based on large-scale population-based studies. Regrettably, China has not paid much attention to trace element status. As a result, the extent of deficiency or overexposure can hardly be evaluated because no studies have been performed on a national level. Therefore, currently, a deeper health-risk evaluation of the health status of pregnant women in China in terms of trace elements cannot be performed. Fortunately, the China Nutrition and Health Survey 2010–2012 (CNHS 2010–2012) can provide trace elements profiles in all population groups in China, including pregnant women. 

The purpose of this study was to obtain the concentrations of 14 serum trace elements from pregnant Chinese women and to determine their reference values according to the recommendations of the International Federation of Clinical Chemistry [11], so as to play a key role in protecting populations at greater risk.

## 2. Materials and Methods

### 2.1. Study Population

The CNHS 2010–2012 is a well-designed nationally representative cross-sectional study using a multistage stratified cluster sampling method. It covered all 31 provinces, autonomous regions, and municipalities directly under the central government throughout China. The whole county was divided into urban and rural regions according to their economic status and social development. The present study was performed based on the CNHS 2010–2012, and we had randomly chosen seemingly healthy pregnant women aged 18–44 years as the study subjects in this study from the 150 monitoring sites around China. 

The CNHS 2010–2012 was conducted in accordance with the principles of the Declaration of Helsinki [12]. All detailed field procedures involving human subjects were approved by the Ethics Committee of the National Institute for Nutrition and Health, Chinese Center for Disease Control and Prevention. All subjects provided consent for their participation in the CNHS 2010–2012 after the nature of the survey was explained.

### 2.2. Sample Collection, Preparation, and Determination

Blood samples were drawn from the antecubital vein by venipuncture into a separation gel vacuum blood collection tubes after a fasting period of 10–12 h in the morning. All blood samples were centrifuged at 1500× *g* for 10 min within 0.5–1 h following collection. Serum samples were then extracted and aliquoted immediately into screw-top cryogenic vials, and stored at −70 °C and subjected to repeated freeze-thaw until analysis. 

In this study, all serum specimens were chosen according to the identification number of the subjects’ vials labeled, taking great care to avoid hemolysis samples. The concentrations of 14 serum trace elements, namely iron (Fe), copper (Cu), zinc (Zn), rubidium (Rb), selenium (Se), strontium (Sr), molybdenum (Mo), manganese (Mn), lead (Pb), arsenic (As), chromium (Cr), cobalt (Co), vanadium (V), and cadmium (Cd), were determined by high-resolution inductively coupled plasma mass spectrometry (HR-ICP-MS) following 1:20 dilutions of 100 μL of serum with diluent containing 0.5% (v/v) HNO_3_. The detailed analytical method has been reported in previous literature [13]. During the determination, accuracy and precision were checked by certified commercial serum reference materials (Clinchek Level-1, Level-2, Recipe, Germany) and samples of manual serum sample with spiked concentrations of certain elements. 

### 2.3. Anthropometric Status

Height and weight were measured according to a standard protocol suggested by the World Health Organization (WHO). Height was measured to the nearest 0.1 cm without shoes, using a portable stadiometer and weight was measured in lightweight clothing to the nearest 0.1 kg with a calibrated beam scale. The anthropometric status for pregnant women was classified into four subgroups according to the cutoff values of body mass index (BMI): Underweight (BMI < 18.5), Normal (18.5 ≤ BMI < 25), Overweight (25 ≤ BMI < 30), and Obesity (30 ≤ BMI) [14].

### 2.4. Statistical Analysis

All calculations for determining reference values were in the light of the guidelines found in the Clinical and Laboratory Standards Institute [15]. We had employed the box plot that was used to identify possible outliers. Outliers were removed according to Dixon’s test [15]. When the D/R ratio was over 1:3, the outliers were excluded from this study, where D is the absolute difference between the extreme value and the values nearest to it, and R is the range of all values. The reference values of 14 serum trace elements were expressed as the median anthinnessd the central 95% reference intervals (P2.5–P97.5). The differences in the concentrations of the serum trace elements between subgroups were further assessed using the Kruskal–Wallis test. To analyze the associations of age, residence, anthropometric status, and pregnancy with respect to serum trace elements, a general linear model factorial analysis was applied with Tukey’s post hoc comparisons. All statistical analyses were performed using SAS 9.3 (SAS Institute, Inc., Cary, NC, USA). The statistical values were considered significantly different at *p* < 0.05.

## 3. Results

### 3.1. Population Characteristics

A total of 1400 pregnant women (average age: 27.0 ± 4.5 years) were randomly selected from the urban and rural regions in the CNHS 2010–2012 (Table 1). The study subjects were further divided into the different groupings by age interval (18–25 years, 26–30 years, and 31–44 years), residence (Urban and Rural regions), anthropometric status (Underweight, Normal, Overweight, and Obesity) and duration of pregnancy (Trimester 1: Gestation weeks < 12 weeks; Trimester 2, 12 ≤ Gestation weeks < 28 weeks; Trimester 3: Gestation weeks ≥ 28 weeks). Of these, the data of pregnant women for age intervals and anthropometric status (63, 4.5%) and for residences and duration of pregnancy (151, 10.8%) was removed for lacking of the relevant information and possible outliers.

### 3.2. Analytical Performances

Table 2 summarizes that the limits of method quantification (LOQs) calculated to the undiluted serum range from 0.02 μg/L for ^208^Pb, ^51^V, and ^111^Cd to 2.2 μg/L for ^77^Se. The comparison of target values with measured values shows sufficient agreement for all trace elements, without significant outliers for samples with lower concentrations. The coefficient variation (CV) % of intra-day precision was from 1.7 for ^56^Fe to 7.5 for ^208^Pb, whereas the CV % of inter-day precision ranged from 1.9 for ^66^Zn to 10.6 for ^208^Pb. In addition, the recoveries calculated by the measured concentrations compared with the certified concentrations of control materials were in the range 89.1% for ^111^Cd and 112.5% for ^208^Pb. In addition, the percentage of trace element values below the LOQs in this study were as follows: 14.8% for the ^55^Mn results, 0.3% for ^208^Pb, 24.3% for ^75^As, 5.8% for ^52^Cr, 5.2% for ^59^Co, and 4.4% for ^51^V.

### 3.3. Reference Values of Trace Elements in Serum

The reference values of 14 serum trace elements for pregnant women from the CNHS 2010–2012 are listed in Table 3 and Table 4. In addition to the results for the total study population, information on groupings classified according to age interval, residence, anthropometric status, and duration of pregnancy is provided. Overall, the median concentration of 14 serum trace elements ranges from 133.7 ng/L for Cd in the lower ng/L range up to 195.5 μg/dL for Cu or 131.5 μg/dL for Fe. Essential trace element concentrations are in a relative small range for Fe (55.8–265.0 μg/dL), Cu (107.0–362.4 μg/dL), Zn (51.8–111.3 μg/dL), and Se (39.9–111.6 μg/L). Other essential trace element concentration ranges are as follows: Mo (1.2–3.6 μg/L), Mn (1.2–8.4 μg/L), Co (57.0–1130.0 ng/L), and V (23.6–2323.1 ng/L). Potential essential trace element concentrations ranges are as follows: Rb (14.0–62.0 μg/dL) and Sr (23.8–104.3 μg/L). By contrast, toxic trace element concentrations are in a larger range for Pb (0.6–9.0 μg/L), As (0.3–5.6 μg/L), and Cd (72.1–595.1 ng/L).

There are some remarkable changes in the 14 serum trace element concentrations in the different groupings. Among different age intervals, the concentrations of serum Zn, Se, As, and Cd significantly increased with increasing age (*p* < 0.05), whereas serum Rb concentration significantly decreased (*p* < 0.05). Among residences, significant differences were observed in serum Se, Sr, Mo, Mn, Pb, As, and Cd concentrations between the urban and rural regions (*p* < 0.05). Among different anthropometric statuses, we found that there was a distinctly difference in serum Fe, Cu, Zn, Se, Sr, Co, V, and Cd concentrations (*p* < 0.05). With respect to pregnancy, serum Fe, Zn, and Se concentrations significantly negatively decreased as the duration of pregnancy advanced, while serum Cu, Sr, and Co concentrations markedly positively increased (*p* < 0.05).

## 4. Discussions

The assessment of essential and toxic elements levels during pregnancy was complicated by the dynamic physiological changes that take place in pregnant women. At present, our study had established reference values of 14 serum trace elements for pregnant women selected from the CNHS 2010–2012. It had examined the remarkable differences of 14 serum trace element reference values in terms of age interval, residence, anthropometric status, and duration of pregnancy. Furthermore, it is worth noting that the 14 trace elements have inconsistent variation in the duration of pregnancy. We found that the concentrations of serum Fe, Zn, and Se significantly decreased, whereas serum Cu, Sr, and Co concentrations progressively elevated with advanced anthropometric status and duration of pregnancy. 

In our study, the reference values of 14 serum trace elements are relatively accurate and reliable, reflecting the real status of serum trace elements in the human body. One possible reason could be that HR-ICP-MS was applied and “tailored” to the serum trace elements at ultra-trace analysis, ensuring low limits of detection for its excellent characteristics [16]. This makes us able to analyze the essential and toxic elements at trace and ultra-levels in a significantly high number of serum samples, producing reliable estimations. Another possible reason is that the representative samples of pregnant women were selected from the CNHS 2010–2012. The concentrations of serum trace elements are reported in the literature for the partial elements investigated, but there are some significant differences in content when compared to analogous studies [17,18,19,20]. In our study, the concentrations of serum Fe, Cu, Zn, Se, Sr, and Mn were 131.5 (55–265.0) μg/dL, 195.5 (107–362.4) μg/dL, 74.0 (51–111.0) μg/dL, 72.2 (39–111.6) μg/L, 45.9 (23–104.3) μg/L, and 2.4 (1.2–8.4) μg/L, respectively, which are in agreement with values reported in some of the previous literature [21,22], but were lower or higher when compared to those reported in other studies [23,24,25,26]. As regards the discrepancy, possible reasons include different environmental factors, different socioeconomic status, different dietary patterns, different lifestyles, and racial differences. 

In addition, prior to deriving conclusions on comparison, several factors were required for consideration. As mentioned, age is the one of the first parameters that can significantly affect the body burden of trace elements [27]. In this study, we observed significant variations in serum trace elements between age intervals. Moreover, the concentrations of serum Se, Sr, Mo, Mn, V, As, Pb, and Cd have significant changes by residence. Therefore, economic factors might play an important role. Similarly, the levels of some serum trace elements are significantly different in different levels of anthropometric status. It was considered that the development and progression of obesity could be involved in the dysregulation of trace element metabolism via the increase in excretion and the decrease in bioavailability or redistribution among various pools [28]. With respect to the influence of pregnancy duration, the reasons might be multifactorial. Plasma volume expansion may explain why most trace elements show a decrease in concentration. However, regarding the increase in the concentration of serum Cu, Sr, and Co, we considered that metabolic changes might cause a certain amount of trace elements to be released into the blood. For example, the increase in serum Cu with the progression of pregnancy could be partly related to the synthesis of ceruloplasmin, a major Cu binding protein, as a result of elevated levels of maternal estrogen. Another potential reason is the decreased biliary Cu excretion induced by the hormonal changes that are typical during pregnancy [29]. Apart from the above reasons, other specific reasons could also include natural background conditions, such as geographical location, climate, the composition of soil, and element concentrations in water and food. 

To the best of our knowledge, there have been no national large-scale population studies of serum trace elements similar to this study. In the absence of such studies, our findings can be used to provide the baseline data on serum trace element concentrations for pregnant Chinese women. There are, however, some limitations that must be noted. Firstly, the sample size only involves 1400 pregnant women and this is relatively insufficient and cannot yield a reliably accurate estimation of pregnant women on a national level. Secondly, the concentrations of some trace elements such as Co, V, Cr, Pb, As, and Cd are at very low levels. Thus, the risk for external contamination has to be seriously considered during the process of blood collection, sample preparation, and determination. Thirdly, it is unclear as to what the status of health of the pregnant women in this study was. Thus, the influence of subclinical infections and inflammation on serum trace elements is difficult to further evaluate. Therefore, our future studies will be directed to overcome these limitations.

## 5. Conclusions

In summary, the results of this study obtained baseline data regarding 14 serum trace element concentrations for pregnant women in China based on the CNHS 2010–2012. This valuable data can help to establish the reference values of 14 serum trace elements and provide clear evidence that all of the selected elements are visibly altered when classified by age interval, residence, anthropometric status, and duration of pregnancy. Furthermore, these findings shall be useful for future research to assess the trace element nutritional status and health risks of environmental metal exposure, and to protect population in China at greater risk.

## Figures and Tables

**Table 1:** Characteristics of pregnant Chinese women selected from the China Nutrition and Health Survey 2010–2012 (CNHS 2010–2012).

Variables	Number	Percent (%)
Total population	1400	100
Age (year)		
18–25	528	39.5
26–30	505	37.8
31–44	304	22.7
Residences		
Urban	586	46.9
Rural	663	53.1
Anthropometric status		
Underweight	78	5.8
Normal	789	59.0
Overweight	388	29.0
Obesity	82	6.2
Pregnancy		
Trimester 1	291	21.8
Trimester 2	555	41.5
Trimester 3	491	36.7

**Table 2:** Selected isotope, limits of quantification (LOQs), measured and certified concentrations of reference materials, coefficient variation (CV) % of intra-day and inter-day and recoveries %.

Isotopes	LOQs (μg/L)	Concentration (μg/L)		CV %		Recoveries %
		Certified/Spiked	Measured	Intra-Day	Inter-Day	
^56^Fe	0.4	407	420	1.7	2.4	103.2
^63^Cu	0.2	1390 ± 210	1426 ± 216	2.9	3.1	102.6
^66^Zn	1.6	1090 ± 160	987 ± 145	1.8	1.9	90.6
^85^Rb	0.2	244	220	2.7	3.1	90.2
^77^Se	2.2	158 ± 31	174 ± 34	6.3	8.4	110.1
^88^Sr	0.03	46	45	3.8	3.4	97.8
^95^Mo	0.15	4.18 ± 1.26	4.4 ± 1.3	4.6	6.2	105.3
^55^Mn	1.5	2.4	2.5	2.4	2.4	104.2
^2^°^8^Pb	0.02	0.4	0.5	7.5	10.6	112.5
^75^As	0.7	19.3 ± 3.8	20.3 ± 4.0	5.5	6.8	105.2
^52^Cr	0.25	6.31 ± 1.26	6.5 ± 1.3	3.6	4.0	103.0
^59^Co	0.06	5.77 ± 1.15	6.3 ± 1.3	2.8	2.9	109.2
^51^V	0.02	12.6 ± 2.6	13.5 ± 2.8	2.7	2.9	107.1
^111^Cd	0.02	5.39 ± 1.08	4.8 ± 1.0	4.4	3.5	89.1

**Table 3:** Reference values ranges of serum major trace elements for pregnant Chinese women from the CNHS 2010–2012.

Variables	Fe (μg/dL)		Cu (μg/dL)		Zn (μg/dL)		Rb (μg/dL)		Se (μg/L)		Sr (μg/L)		Mo (μg/L)	
	M_0_	P2.5–P97.5	M_0_	P2.5–P97.5	M_0_	P2.5–P97.5	M_0_	P2.5–P97.5	M_0_	P2.5–P97.5	M_0_	P2.5–P97.5	M_0_	P2.5–P97.5
Total	131.5	55.8–265.0	195.5	107.0–362.4	74.0	51.8–111.3	22.3	14.0–62.0	72.2	39.9–111.6	45.9	23.8–104.3	1.8	1.2–3.6
Age (year)														
18-25	127.0 ^a^	55.6–247.0	196.0 ^a^	102.5–356.0	72.8 ^a^	51.3–107.4	22.4 ^a^	14.0–71.2	68.3 ^a^	37.3–107.5	47.8 ^a^	24.1–108.6	1.8 ^a^	1.2–3.5
26-30	138.0 ^a^	57.0–271.0	195.9 ^a^	109.0–360.5	75.2 ^b^	54.5–114.0	22.4 ^a^	14.4–61.0	75.0 ^b^	42.0–112.0	43.5 ^a^	24.0–100.5	1.8 ^a^	1.2–3.7
30-44	132.0 ^a^	56.0–273.0	194.0 ^a^	113.0–373.7	73.4 ^a,b^	51.6–110.0	21.6 ^b^	13.6–48.8	73.0 ^b^	41.0–118.0	45.0 ^a^	23.0–100.5	1.7 ^a^	1.1–3.5
Residences														
Urban	135.0 ^a^	56.9–250.7	194.0 ^a^	108.0–302.0	74.8 ^a^	51.3–110.6	22.1 ^a^	14.0–43.2	75.2 ^a^	42.5–111.5	40.2 ^a^	23.0–97.3	1.7 ^a^	1.2–3.4
Rural	127.6 ^a^	55.0–276.3	196.0 ^a^	107.0–426.0	73.2 ^a^	52.1–111.8	22.7 ^a^	14.0–80.4	69.5 ^b^	38.0–110.0	51.0 ^b^	24.3–109.8	1.8 ^b^	1.2–3.8
Anthropometric status						.								
Underweight	152.5 ^a^	82.7–281.4	193.0 ^a^	99.7–624.9	82.4 ^a^	58.0–150.3	24.9 ^a^	15.1–70.6	75.2 ^a^	52.2–144.1	43.4 ^a^	24.5–109.8	1.8 ^a^	1.2–3.9
Normal	136.1 ^b^	57.0–265.3	193.2 ^a^	104.7–343.5	74.6 ^b^	53.4–110.5	22.4 ^a^	14.1–67.2	73.6 ^a^	40.6–111.9	44.9 ^a,b^	23.0–101.9	1.8 ^a^	1.2–3.7
Overweight	118.8 ^c^	53.9–242.6	199.7 ^a^	117.0–378.4	72.7 ^c^	50.4–106.8	21.8 ^a^	13.9–60.9	68.2 ^b^	38.6–105.3	48.9 ^b,c^	24.9–109.9	1.8 ^a^	1.1–3.4
Obesity	110.6 ^d^	48.8–222.7	212.2 ^b^	116.9–292.4	69.9 ^d^	50.9–90.8	21.5 ^a^	13.7–31.0	66.8 ^b^	37.3–99.5	50.1 ^c^	25.9–100.4	1.7 ^a^	1.1–2.8
Pregnancy														
Trimester 1	150.0 ^a^	70.9–285.6	157.5 ^a^	89.7–366.0	83.2 ^a^	57.7–129.5	22.9 ^a^	13.7–80.9	77.6 ^a^	44.0–112.0	43.2 ^a^	22.6–102.0	1.8 ^a^	1.2–3.6
Trimester 2	135.0 ^b^	59.2–261.3	198.6 ^b^	122.6–343.5	73.0 ^b^	53.4–102.6	21.9 ^a^	14.5–62.0	76.0 ^a^	42.5–112.0	42.2 ^a^	22.5–92.5	1.8 ^a^	1.2–3.5
Trimester 3	110.3 ^c^	52.2–260.7	208.2 ^c^	121.0–375.0	71.0 ^c^	49.4–110.0	22.4 ^a^	13.9–52.2	65.0 ^b^	38.0–105.0	52.8 ^b^	26.0–119.5	1.8 ^a^	1.1–3.7

M_0_: Median Concentration. A general linear model was performed with Least Squares Means post hoc analysis to compare the effect of age. Values not sharing the same superscript letter (a–d) denote a significant difference between subgroups, *p* < 0.05.

**Table 4:** Reference values ranges of serum major trace elements for Chinese pregnant women in the CNHS 2010–2012 (continue).

Variables	Mn (μg/L)		Pb (μg/L)		As (μg/L)		Cr (ng/L)		Co (ng/L)		V (ng/L)		Cd (ng/L)	
	M_0_	P2.5–P97.5	M_0_	P2.5–P97.5	M_0_	P2.5–P97.5	M_0_	P2.5–P97.5	M_0_	P2.5–P97.5	M_0_	P2.5–P97.5	M_0_	P2.5–P97.5
Total	2.4	1.2–8.4	1.9	0.6–9.0	1.1	0.3–5.6	835.6	219.8–4287.7	337.9	57.0–1130.0	193.2	23.6–2323.1	133.7	72.1–595.1
Age (year)														
18–25	2.4	1.3–9.0	1.9	0.6–8.2	1.1 ^a^	0.3–7.3	879.2	248.9–4295.3	353.2	68.5–1129.8	172.4	29.7–2130.5	126.2 ^a^	71.9–614.3
26–30	2.4	1.2–7.7	2.0	0.6–8.9	1.1 ^a^	0.4–4.5	798.0	205.0–3688.0	333.6	48.5–1085.5	208.3	17.5–2323.0	140.0 ^b^	73.0–549.0
31–44	2.3	1.1–8.4	2.0	0.5–10.5	1.3 ^b^	0.4–5.2	799.0	211.0–5546.0	333.0	89.0–1250.5	215.0	20.0–2486.0	137.5 ^b^	71.0–598.5
Residences														
Urban	2.2 ^a^	1.1–8.4	2.1 ^a^	0.7–8.9	1.3 ^a^	0.4–4.7	845.5	79.0–5395.0	338.0	52.0–1250.6	218.4 ^a^	18.5–2625.5	139.3 ^a^	72.4–624.8
Rural	2.5 ^b^	1.2–7.8	1.8 ^b^	0.5–9.2	1.0 ^b^	0.3–7.5	827.4	297.9–3387.0	339.0	73.9–1041.6	174.9 ^b^	31.0–1797.0	129.5 ^b^	71.3–563.9
Anthropometric status														
Underweight	2.2 ^a^	1.2–6.5	2.2 ^a^	0.7–9.2	1.5 ^a^	0.4–4.4	909.0 ^a^	317.4–3135.6	234.0 ^a^	21.6–917.0	125.0 ^a^	13.6–2000.3	150.3 ^a^	72.8–426.2
Normal	2.4 ^a^	1.2–9.2	1.9 ^a^	0.6–10.1	1.1 ^a^	0.4–5.4	815.2 ^a^	236.0–4587.5	317.9 ^b^	52.9–1083.3	201.2 ^b^	25.3–2485.7	137.8 ^a^	72.6–660.7
Overweight	2.4 ^a^	1.2–7.0	1.9 ^a^	0.5–7.0	1.0 ^a^	0.3–5.7	835.5 ^a^	158.8–3913.2	391.0 ^c^	93.6–1219.9	209.3 ^b^	22.2–1965.6	125.5 ^b^	68.2–488.0
Obesity	2.3 ^a^	1.4–9.2	1.7 ^a^	0.6–8.1	1.3 ^a^	0.4–7.3	881.9 ^a^	263.6–3320.8	394.3 ^c^	112.4–1113.1	182.0 ^b^	30.9–1885.6	115.3 ^b^	71.9–290.7
Pregnancy														
Trimester 1	2.4	1.1–9.2	2.1 ^a^	0.7–9.2	1.0 ^a^	0.4–4.7	888.0 ^a^	219.8–4287.7	217.6 ^a^	26.0–1093.9	206.8 ^a^	22.2–2401.3	136.5 ^a^	71.9–618.3
Trimester 2	2.3	1.1–8.4	1.8 ^b^	0.6–6.5	1.1 ^a^	0.3–6.4	789.5 ^a^	248.9–4295.3	313.0 ^b^	45.2–1083.0	178.4 ^a^	23.6–2564.2	133.2 ^a^	71.9–456.3
Trimester 3	2.5	1.4–7.7	2.0 ^a^	0.6–12.3	1.3 ^a^	0.3–5.0	837.0 ^a^	205.0–3688.0	432.0 ^c^	136.2–1206.0	203.0 ^a^	23.7–1965.6	134.0 ^a^	74.0–757.0

M_0_: Median Concentration. A general linear model was performed with Least Squares Means post hoc analysis to compare the effect of age. Values not sharing the same superscript letter (a–d) denote a significant difference betweenin subgroups, *p* < 0.05.
